# Antihypothyroid Effect of Salidroside

**DOI:** 10.3390/molecules27217487

**Published:** 2022-11-02

**Authors:** Nazym K. Korbozova, Nataliya O. Kudrina, Nataliya A. Zhukova, Alexander E. Grazhdannikov, Irina V. Blavachinskaya, Gulnaz A. Seitimova, Timur E. Kulmanov, Tatyana G. Tolstikova, Nina V. Terletskaya

**Affiliations:** 1 Faculty of Biology and Biotechnology and Faculty of Chemistry and Chemical Technology, Al-Farabi Kazakh National University, Al-Farabi Av., 71, Almaty 050040, Kazakhstan; 2 Institute of Genetic and Physiology, Al-Farabi Av., 93, Almaty 050040, Kazakhstan; 3 N.N. Vorozhtsov Novosibirsk Institute of Organic Chemistry, Siberian Branch of Russian Academy of Science, 630090 Novosibirsk, Russia

**Keywords:** *Rhodiola semenovii* Boriss., salidroside, thyroid, antihypothyroid effect

## Abstract

In terms of prevalence, thyroid pathology, associated both with a violation of the gland function and changes in its structure, occupies one of the main places in clinical endocrinology. The problem of developing low-toxic and highly effective herbal preparations for the correction of thyroid hypofunction and its complications is urgent. Salidroside is a glucoside of tyrosol, found mostly in the roots of *Rhodiola* spp., and has various positive biological activities. The purpose of this study was to study the antihypothyroid potential of salidrosid-containing extract from *R. semenovii* roots, which was evaluated on a mercazolyl hypothyroidism model. We showed that extract containing salidroside is a safe and effective means of hypothyroidism correction, significantly reducing (*p* ≤ 0.001) the level of thyroid-stimulating hormone and increasing the level of thyroid hormones. The combined use of *R. semenovii* extract with potassium iodide enhances the therapeutic effect of the extract by 1.3-times.

## 1. Introduction

In terms of prevalence, thyroid pathology, associated with both a violation of the function of the gland and a change in its structure, occupies one of the main places in clinical endocrinology [1,2]. Currently, the greatest prevalence and clinical significance is primary hypothyroidism, caused by a defect in the biosynthesis of the hormones T4 and T3, due to disorders in the thyroid gland. The currently used principles of correction for hypothyroidism practically do not depend on pathogenesis and are reduced to hormone replacement therapy. Insufficient and inadequate replacement therapy with thyroid hormones contributes to the progression of coronary heart disease, impaired reproductive function, depression, and an overdose is dangerous for the development of myocardial dystrophy with atrial fibrillation [2]. Thyroid hormones are critical for normal brain development, for orchestrating the developmental processes of neurogenesis, migration, synaptogenesis, and myelination [3,4].

Plant extracts, or their individual substances (metabolites) due to their chemical diversity, significantly expand the possibilities for creating effective drugs [5]. In this regard, the problem of development of low-toxic and highly effective drugs for the correct ion of hypothyroidism is relevant. With the entry into force of the Nagoya Protocol in 2021, the search for new internal effective resources of medicinal plants has become particularly relevant for each country [6]. Plants of the *Crassulaceae* family are natural accumulators of organic acids. The main biologically active components in the genus are rhodiophlafonozides, in particular salidroside, which, according to the literature, stimulates the immune system, enhances cognitive abilities, helps to resist stress, and relieves symptoms of chronic fatigue, as well as contributing to an increase in nonspecific immunological reactivity of the body [7,8,9,10]. Salidroside is produced by a three-step pathway in plants with tyrosol as an intermediate molecule, which can be biotransformed into many metabolites. These metabolites, together with a certain amount of salidroside, may be responsible for various pharmacological functions [11].

*Rhodiola rosea* L., a popular medicine plant, has also been proposed for the alternative treatment of symptoms of short-term hypothyroidism in patients who require hormone withdrawal [12]. However, *R. rosea* is a rare species whose resource intensity is currently catastrophically reduced [13]. Therefore, it is necessary to comprehensively study the biological and pharmacological activity of other representatives of this family and, in particular, *Rhodiola semenovii* (Regel & Herder) Boriss. This species has a long record of usage in traditional medicine in Central Asian countries as a source of tonic, adaptogenic, anti-inflammatory, choleretic, hypoglycemic, and antioxidant qualities [14,15,16,17]. However, there are now very few literature sources devoted to the research of *R. semenovii’s* metabolic profile, including medicinally useful components in this species’ plants. We assume that salidrosid-containing extract from *R. semenovii* as *R. rosea* can contribute to the normalization of thyroid functions. All this makes these studies particularly relevant. The thyroid hormone system can be affected by insufficient iodide intakes. The global health problem in the world, in particular, in some regions of Kazakhstan, is iodine deficiency, which is an important trace element for the synthesis of thyroid hormones [18,19]. Thus, the purpose of this study was to examine and implement the extract of the plant *R. semenovii* and its combination with KI in the correction of experimental hypothyroidism.

## 2. Results

### 2.1. Determination of Salidroside in R. semenovii Root Extract

**Figure 1:**
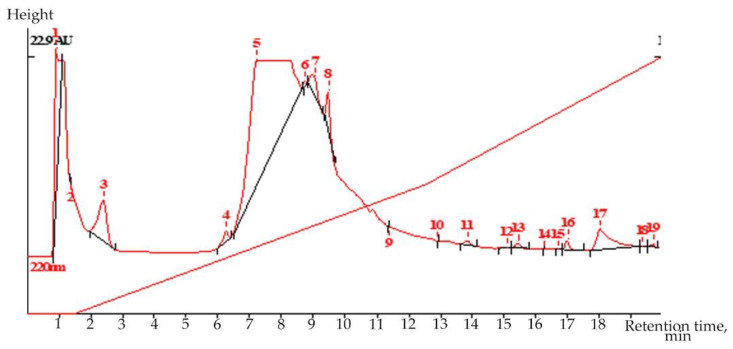
Chromatogram of salidroside standard and extract of *Rhodiola semenovii* roots: Dry residue content in extract 4: (100 × 0.4367)/42.8450 = 1.02%; Tyrosol content in dry part of extract: (100 × 27.442 × 3.3/20)/(80.025 × 169.8/3) = 0.0999% (calibr. sol. 2–220 nm); Salidroside content in the dry part of extract: 0.217%.

Liquid chromatography (LC) is a highly selective, but extremely flexible and very sparing, method for isolating secondary metabolites from plants with weak chromophores. Liquid chromatography revealed the presence of the valuable drug glycoside–salidroside (0.217%) in root extract in the flowering phase of *R. semenovii* plants (Figure 1 and Supplementary Materials Figures S1 and S2).

Volume 20.0 µL; COLUMN ProntoSil-120-5-C18 AQ; Size 2.0 × 75 mm; Part size 5.0 µm;
Comments: Extr 4(upar) in MeOH-H_2_O; MeOH-H_3_PO_4_ 1:9-11:9-1:0 300:2500:4000;
Flow 200.00 mkl/min; Temperature 35.0 °C; Pressure 5.0 MPa

### 2.2. The Effect of Dry Extract of Rhodiola semenovii on the Level of Thyroid Hormones and Thyroid Tissue in Rats

**Figure 2:**
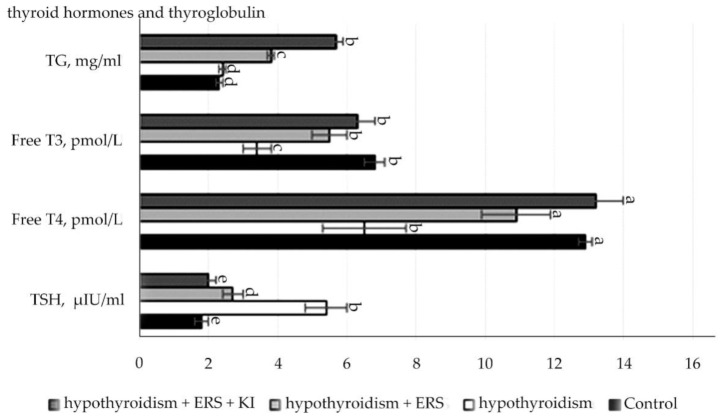
Indicators of the level of thyroid hormones and thyroglobulin on the hypothyroidism model after correction with *R. semenovii* extract, alone and in combination with potassium iodide. Values presented are means (±SD). Different letters above the bars represent significant differences at *p* ≤ 0.05.

Analysis of the data presented in Figure 2 showed that experimental hypothyroidism had statistically significant changes (*p* ≤ 0.001): increase in the level of TSH, decrease in the levels of both hormones, and thyroxine free and triiodothyronine free.

There were statistically significant differences (*p* ≤ 0.001) in the concentration of TSH levels in the group with experimental hypothyroidism in relation to those in the control group, as well as in the experimental groups with correction of hypothyroidism with the extract of *R. semenovii* alone and corrected with extract of *R. semenovii* in combination with potassium iodide in relation to the ones in the group with experimental hypothyroidism.

In the control group, there was a statistically significant decrease of 2-times (at *p* ≤ 0.001) in the concentration of free T4 in comparison with that in the group with modeling of experimental hypothyroidism. While conducting the study in groups with the extract from the root of *R. semenovii* correction with the addition of potassium iodide, there was also a free T4 increase by 2-times. 

Statistically significant values (*p* ≤ 0.001) were also noted for the concentration of free T3 in hypothyroidism, which decreased by 2-times compared to those in the control group. At the end of treatment, free T3 increased in the experimental groups with the correction of hypothyroidism with extract of *R. semenovii* and correction with extract of *R. semenovii* in combination with potassium iodide in relation to that in the group with experimental hypothyroidism.

**Figure 3:**
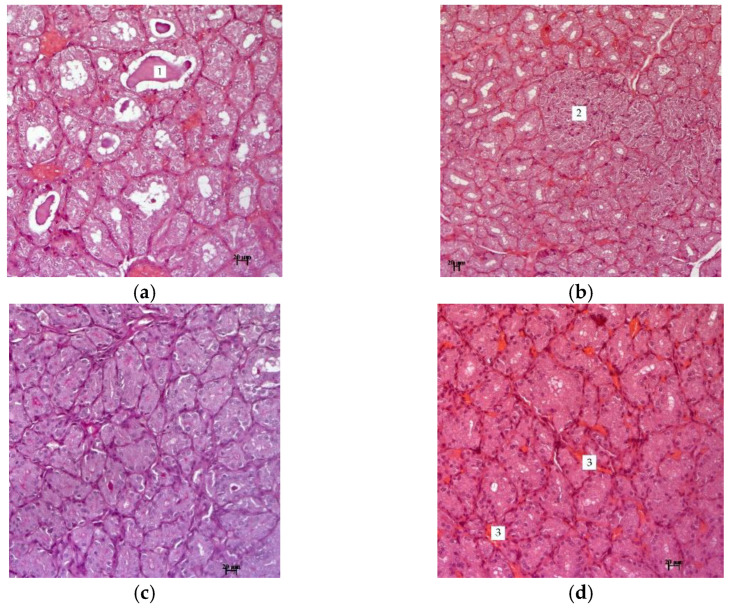
Changes in the tissues of the thyroid gland of rats with mercazolyl hypothyroidism and its treatment with an extract of *R. semenovii*: (**a**) hypothyroidism—a small amount of colloid is represented by PAS (periodic acid Schiff reaction)—positive drops that do not fill the lumen of the follicles, which indicates a decrease in secretion by thyrocytes, coloration of PAS—hematoxylin—orange G, (**b**) focal nodular formations in the form of solid follicle-like structures, without colloid, formed by proliferating epithelium from follicles, coloration of hematoxylin and eosin, (**c**) treatment with *R. semenovii*, absence of fibrous and nodular formations, improvement in thyrocytes structure, vascular plethora, coloration of PAS—hematoxylin—orange, (**d**) treatment with *R. semenovii* extract in combination with KI, homogeneous cytoplasm of thyrocytes, absence of nodules in the stroma, preservation of plethora, coloration of hematoxylin and eosin. 1—PAS-positive drops; 2—follicle-like structures; 3—full-blood vessels; scale bar = 200 µm.

Figure 3 shows photographs of thyroid tissue in experimental hypothyroidism and after treatment with the described methods. 

The research results indicate that the thyroid gland of hypothyroid animals is formed by numerous follicles of various sizes. The bulk of the follicles are formed by tyrocytes, which are located on the basement membrane in one layer. They have a cubic shape and spherical nuclei. The cytoplasm of thyrocytes has an enlightened structure in the perinuclear zone (Figure 3). In the thyroid gland of all animals, focal nodules in the form of solid structures are observed, which are characterized by the proliferation of the epithelium from follicles with the formation of follicle-like formations without a colloid. 

**Figure 4:**
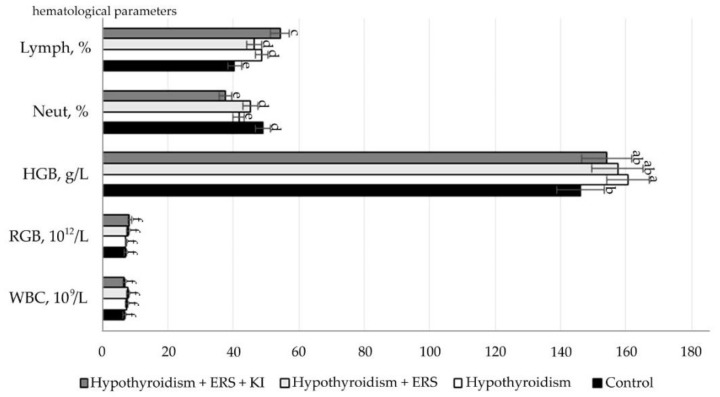
Hematological parameters of rats with hypothyroidism after correction with *R. semenovii* extract alone and that in combination with potassium iodide. Values presented are means (±SD). Different letters above the bars represent significant differences at *p* ≤ 0.05.

The results of histological studies in groups with *R. semenovii* extract correction, in contrast to the group with experimental hypothyroidism, show no pronounced negative dynamics from the morphology of the thyroid gland. There were also no signs of fibrosis in inter-follicular tissue. In the gland tissue of all animals, there is a well-developed network of blood vessels and improvement in the structure of thymocytes (Figure 4). The cytoplasm of the epithelium is homogeneous, unchanged, and there are no nodal solid formations.

### 2.3. Effect of Dry Extract of Rhodiola semenovii on Body Weight and Mass Coefficients of Rat Organs on an Experimental Model of Mercazolyl Hypothyroidism

The results of the measurement of body weight and mass coefficients of rat organs at hypothyroidism after correction with extract of *R. semenovii* alone and in combination with potassium iodide are given in Table 1.

As follows from the data presented in Table 1, in the groups with correction of hypothyroidism ERS and with correction of hypothyroidism ERS + KI, animals body weight is statistically significantly (*p* ≤ 0.001) lower in relation to that in the group of animals with experimental hypothyroidism. A statistically significant (*p* ≤ 0.001) increase in liver mass of 2-times in the studied group of animals with experimental hypothyroidism compared with that in the control group was also revealed. In the groups with ERS correction of hypothyroidism and correction of hypothyroidism ERS + KI, this indicator was lower in relation to that in the studied group of animals with experimental hypothyroidism.

Statistically significant changes (*p* ≤ 0.001) were noted for the increase in the thyroid gland mass in the studied group with experimental hypothyroidism in relation to that in the control group. 

As for the remaining organs of all group animals, statistically significant changes (*p* ≤ 0.001) were not detected.

### 2.4. The Effect of Dry Extract of Rhodiola semenovii on the Peripheral Blood Parameters of Rats and Liver Function

**Figure 5:**
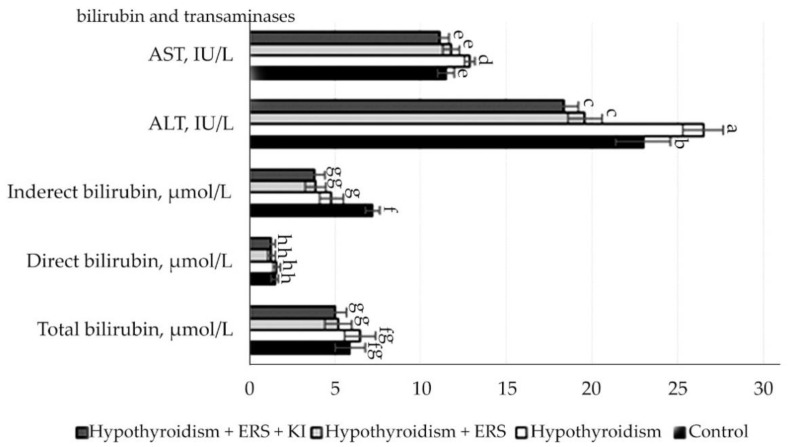
Indicators of the level of bilirubin, transaminases on the hypothyroidism model after correction with *R. semenovii* extract alone and the one in combination with potassium iodide. Different letters above the bars represent significant differences at *p* ≤ 0.05.

The obtained study results of the correction effect on peripheral blood parameters are presented in Figure 5.

As follows from the data presented in Figure 2, after corrective application of extract *R. semenovii* alone and in combination with potassium iodide, significant changes in hematological parameters were not revealed. 

Statistically significant differences (*p* ≤ 0.001) were noted in the relative neutrophil content in animals in the studied group with experimental hypothyroidism without treatment in relation to that in the group with extract of R. *semenovii* correction. 

Statistically significant differences (*p* ≤ 0.001) were also identified in the relative content of lymphocytes in animals in the group with extract of *R. semenovii* correction and of those in groups corrected by extract of *R. semenovii* in combination with potassium iodide in relation to figures in the studied group with experimental hypothyroidism.

According to the results presented in Figure 5 indicators of the level of bilirubin and its fractions, transaminases in rats in comparison with control, and those in experimental groups had statistically significant changes, but all these changes were within the physiological norms.

Statistically reliable (*p* ≤ 0.001) changes were observed in the transaminases ALAT and ASAT in relation to the control group of animals, which included the increase in their activity in the studied group with experimental hypothyroidism and decrease when corrected with pure extract of *R. semenovii* and in combination with potassium iodide.

### 2.5. The Effect of Dry Extract of Rhodiola semenovii on the Parameters of Protein and Lipid Metabolism in Rats

As follows from the data presented in Table 2, the visible significant changes in lipid metabolism between compared animals in the control and experimental groups were within the physiological norm.

The results of changes in protein and carbohydrate metabolism are presented in Table 3.

As revealed, experimental hypothyroidism was accompanied by disturbances in protein metabolism: the total protein content in the serum of rats with experimental hypothyroidism in relation to that in the control group showed statistically significant changes. Further, in groups with extract of *R. semenovii* correction and the one with extract of *R. semenovii* in combination with potassium iodide in relation to the group containing experimental hypothyroidism, statistically significant changes were observed. 

Albumin and urea concentrations also showed statistically significant changes (*p* ≤ 0.001). Results suggest statistically significant differences (*p* ≤ 0.001) in uric acid content in groups with pure extract of *R semenovii* correction and the one with extract of *R. semenovii* in combination with potassium iodide in relation to the group with experimental hypothyroidism.

According to the creatinine concentration, which was correlated with the mass of muscle tissue and glomerular filtration of the kidneys, statistically significant changes in the blood were revealed (*p* ≤ 0.001). This was noted in animals with experimental hypothyroidism in comparison with the control group of animals with experimental hypothyroidism in relation to other ones with correction of the *R. semenovii* extract, both alone and in combination with potassium iodide. 

Changes in the rats’ kidneys were reversible, as evidenced by urea levels in experimental groups with correction of hypothyroidism with extract of *R. semenovii* alone and in combination with potassium iodide in relation to those in the group with experimental hypothyroidism.

In the blood of animals, statistically significant values (*p* ≤ 0.001) were revealed for glucose concentration in the group with experimental hypothyroidism in comparison with that of the control group, as well as in the group with extract of *R. semenovii* correction in combination with potassium iodide in relation to the group with experimental hypothyroidism and the one with correction of extract of *R. semenovii*.

## 3. Discussion

The phytochemical spectrum of *Crassulaceae* has various biologically active phytochemical components that may have the pharmacological effects and therapeutic properties in various diseases, as well as enhance each other’s effectiveness [7]. *Rhodiola* genus plants are rich in phenolic compounds, such as phenylethanoids, phenylpropanoids, phenolic acids, flavonoids and proanthocyanidins, anthraquinones, as well as carbohydrates, organic acids, essential oils, sterols, terpenoids, lipids (fats, waxes) [10,20,21]. At the same time, quantitative GC-MS analysis *in R. semenovii* previously revealed certain regularities in the accumulation of fatty acids, phenols, ketones, terpenes, lactones, and ubiquinones in plant tissues during the growing season [10]. Yousef et al. discovered cyanogenic glucosides rhodiocyanoside A and lotaustralin, proanthocyanidin oligomers, and polymers in the *R. semenovii* root [21]. However, the major component is salidroside, an important phenylpropanoid glycoside, and the content of this compound is often used as one of the main criteria for the evaluation of the quality of crude extracts from *Rhodiola* plants [22,23,24,25]. At the same time, our study showed a high content of salidroside, not only in the root, but also in the shoot of *R. semenovii* [10].

Complex treatment with thyroids is widely used in medicine. However, due to the high oxidative activity of iodine, it has serious drawbacks. Various biocompatible polymers are used to enhance the binding capacity of molecular iodine. Strong complexes that can be implemented in medicine are formed with the help of them [26]. As our research showed, the use of plant extract of *R. semenovii* in combination with KI can weaken the interaction of iodine with proteins in the gastrointestinal tract without any significant decrease in pharmacological activity. Accumulating in the thyroid gland, iodine is an important component for the production of thyroid hormones to regulate the functioning of the thyroid gland.

Thus, the obtained data resulting from the modeling mercazolyl hypothyroidism demonstrate a three-fold increase in the thyroid gland and histopathological changes in follicles, thyrocytes, and stroma. This confirms the data of Nishihara et al. [27], observed in induced hypothyroidism, for the phenomenon of diffuse lymphoplasmocytic infiltration with separate foci of fibrosis and enlargement of the thyroid gland, also observed by Torlak et al. [28]. They noted hyaline thickness of the blood vessel wall, necrotic follicles, pronounced inflammatory reaction, and detachment of necrotic cells in the follicles. In our experimental treatment of rats by plant extract of *R. semenovii* in combination with KI, positive results in normalization of the structure of thymocytes, with the absence of signs of fibrosis in the interfollicular tissue were clearly shown on histological preparations. A well-developed network of blood vessels, the uniformity of the cytoplasm of the epithelium, and the structure of the thyroid gland as a whole were noted as well. Hypothyroidism causes a decrease in the number of red blood cells, hematocrit, and hemoglobin concentration, which results in severe anemia [29]. Similar results are partially confirmed in the presented data. There is a decrease in the number of red blood cells and platelets. Erythrocytes of blood contain hemoglobin, carry oxygen, and a decrease in erythrocytes leads to anemia. Any decrease in platelets leads to fragility of the blood vessels. In the thyroid damage model, most significant changes were observed in animals with iodine deficiency, namely a significant increase in Hb, Hct, MCV, and a visible decrease in MCHC, WBC, and PLT, rather than sulfadimethoxine, for which only a significant decrease in WBC was recorded. This indicates that such an effect on the blood (leukopenia) may result from either hypersensitivity reactions or metabolites of hydroxylamine or nitrososulfodimethoxine [30]. However, with the *R. semenovii* extract correction, the level of erythrocytes was increased, which indicates the restoration of erythropoiesis. It is suggested that such restoration promotes better oxygen supply to different types of body tissue.

Hypothyroidism causes some decrease in cerebral blood flow [31]. It also promotes a reduction in the expression of glucose transporter GLUT1 to the blood–brain barrier [32]. This can lead to a decrease in glucose input into the brain. According to the results obtained on animal blood counts, statistically significant values for glucose concentration were found in the group with experimental hypothyroidism in relation to the control group, as well as in the group with extract of *R. semenovii* correction in combination with potassium iodide in relation to those in the group with experimental hypothyroidism and the group with the *R. semenovii* extract correction.

In addition, insulin resistance is usually supposed with patients with hypothyroidism [33], which can trigger a decrease in glucose intake by neurons. The glucose increase in the body leads to hyperglycemia, which is the destruction of kidney tissue, heart, blood vessels, and nervous system. Damage to the brain and nerve cells can result from the reduction in glucose content [34]. Dramatic violation of biochemical parameters of renal function is connected with the state of hypothyroidism [35].

According to the results of the experiment, the simulated hypothyroidism was accompanied by disturbances in protein metabolism: the total protein content in the serum of rats with experimental hypothyroidism slightly exceeded the normal values in relation to that in the control group. That confirms the inflammatory process in the body. Clinical diagnosis of diseases and lesions usually assess the structural integrity of the liver by monitoring the state of serum AST and ALT activity [36], including enzymatic activity of AST (Aspartate aminotransferase), ALT (Alanine aminotransferase), and sensitive serological indicators of hepatotoxicity [37,38]. This is partially confirmed in our research. 

Thyroid hormones (thyroxine T4 and triiodothyronine T3) are known to be involved in the regulation of various body functions, including lipid and carbohydrate metabolism, cholesterol metabolism, oxygen consumption, and some physiological processes [39]. The essential role in all major metabolic pathways is performed by thyroid hormones, thyroxine (T4), and triiodothyronine (T3) [40]. TG (thyroglobulin) is also an important factor in metabolism regulation of lipids, cholesterol, and glucose in the liver [41]. According to the results of the study, differences were found in the total cholesterol increase in the group with experimental hypothyroidism in relation to that in the control group. They were also in its decrease in the correction groups with *R. semenovii* extract in combination with potassium iodide in relation to that in the group with experimental hypothyroidism. Total cholesterol (cholesterol) is a fat-like substance, which is necessary for the normal functioning of cells, digestion of food, and the creation of many hormones in the body.

As the data show, changes in the kidneys of rats were reversible, as evidenced by urea levels in experimental groups while correction of hypothyroidism with *R. semenovii* extract. Serum creatinine level is elevated and the glomerular tubule filtration rate is reversibly reduced (GFR), which affects kidney function and structure in patients with obvious hypothyroidism [42]. These are hyperlipidemia and hypercholesterolemia that can occur as a result of increased mobilization of fat stores in the body and with an increased level of thyroid-stimulating hormone induced by hypothyroidism. Low thyroxine levels in hypothyroid animals not only result in the increase in thyrotropin levels during pituitary secretion, but also stimulate corticotrophin, an adrenal steroid that can induce lipid mobilization by blocking the endocrine axis [43].

Clinical trials indicate that a wide range of herbal preparations and plant extracts or natural isolated compounds have a beneficial therapeutic effect on blood flow [44]. In particular, phenols and flavonoids, have, among other things, an antihypertensive effect. [45,46]. 

Therefore, according to the statistical data processing, the phytoextract of *R. semenovii* is not toxic according to the mass coefficients of rat organs, indicators of shaped blood elements, biochemical data of liver function, protein, and lipid metabolism. Experimental therapy of mercazolyl hypothyroidism with *R. semenovii* extract is recognized as effective. Morphological, biochemical parameters, and analysis of thyroid function suggest a statistically significant (*p* ≤ 0.001) decrease in thyroid-stimulating hormone levels and increase in thyroid hormone levels. After treatment of hypothyroidism in groups with the use of *R. semenovii* extract according to the results of hormone indicators, free T4 increased by 2-times, and with *R. semenovii* extract correction in combination with potassium iodide, it showed positive improvement. Statistically significant values were also noted for the concentration of free T3 in hypothyroidism, which decreased by 2-times in relation to those in the control group. At the end of treatment, free T3 increased in experimental groups with correction of hypothyroidism with *R. semenovii* extract. Correction with *R. semenovii* extract in combination with potassium iodide also showed noticeable depositories. 

## 4. Conclusions

Based on the analysis, the data obtained from morphological, biochemical parameters, and analysis of the thyroid function of the use of salidroside from *R. semenovii* plant root extract have shown its safety and efficacy in correction and stabilization of hypothyroid states in the experimental model of mercazolil hypothyroidism.

## 5. Materials and Methods

### 5.1. Plant material and Extraction

The roots of the plant *R. semenovii* Boriss. were collected in the foothills of the Trans-Ili Alatau at an altitude of 2350 m above sea level—at the station “Alpine Rose”—and identified by the Botanical Garden at the Institute of Botany and Phytointroduction in Almaty, Kazakhstan. The voucher specimen number of the sampled plant is 3885. 

The air-dried roots were cut into small pieces and stored at room temperature for one week. The dried samples were ground using a mill (SM 100, “Retsch”, Haan, Germany) to obtain coarse powder. The air-dried powdered roots (2.9 kg) of *R. semenovii* were exhaustively extracted by maceration for 72 h at room temperature with 70% ethanol in a 1:8 ratio until complete exhaustion. The ethanolic extract was concentrated under reduced pressure using a rotary vacuum evaporator at a temperature not exceeding 45 °C giving 300 g of dry residue.

### 5.2. Determination of Salidroside in R. semenovii Root Extract

The water–alcohol extracts of *R. semenovii* roots were kept at a temperature of +40–50 °C. The extracts were evaporated to a constant weight of dry residues (within 10–12 h). Chromatographic experiments were performed on a Milichrom-A-02 liquid chromatograph (“EcoNova” JSC, Novosibirsk, Russian Federation) with a column 75 × 2.0 mm packed with a ProntoSIL-120-5-C18 sorbent. Then, 20 μL of the dried extract solution (solvent MeOH-H_2_O, 2:1) was collected in the needle of the sample injection device; the sample was injected into the device. Chromatography was performed in gradient mode. The eluent feed rate was 200 µL/min. Detection was undertaken at a wavelength of 220 and 280 nm. Gradient composition: 300 µL methanol-0.05 N H_3_PO_4_ mixture (1:9, *v*/*v*)—2200 µL methanol-0.05 N H_3_PO_4_ mixture (11:9, *v*/*v*); further, a gradual increase in the concentration of methanol to pure. The duration of the analysis was 20 min. The retention time of tyrosol was 6.5 to 7.5 min. The limit of detection of tyrosol in the described experiments is 0.005% (for the dry part of the extract). The location of the tyrosol peak on the chromatogram was determined by the addition method. The concentration of tyrosol was determined in comparison with the chromatogram of pure tyrosol (tyrosol produced by NIOCH SB RAS, SOV 98% by GLC). No ion chromatograms were recorded. The dry residue content in the extract was calculated by the formula:

1: (100 × m dry residue)/m extract 1

The tyrosol content in the dry part of extract 1 was calculated by the formula:

1: 100 × S tyrosol on chrom. of extr. × C tyrosol in calibr. sol. / S tyrosol on chrom. of calibr. sol. × C tyrosol in calibr. sol.

The content of salidroside in the dry part of extract 2 was calculated by the formula:

C tyrosol in the dry part of extr.1 × 2.17

### 5.3. Animals

All preclinical experiments with animals were performed after positive permission of ethical commission of the RSE “Institute of Human and Animal Physiology” CS MES RK. Validity period is from 1 October 2020 to 31 December 2022. Protocol number 07-05/71 dated 18 June 2020. White laboratory rats weighing 200–250 g were in standard conditions on the vivarium diet. Further, male drain CD 1 mice weighing 25–35 g (8 individuals in a cage) and male rats of the Vistar line weighing 220–250 g. Animals (rats) were kept in cages in groups of 5 individuals. Sawdust was used as a litter. The air temperature in the premises of the vivarium was maintained in a range of 18–200 °C at a relative humidity of 60–70%. Animals received proper care daily. Animals were obtained from the vivarium of the Institute of Cytology and Genetics of the Siberian Branch of the Russian Academy of Sciences and were kept in special installations for SPF vivarium in IVC systems of individually ventilated cells, SK-MVCS-70RRD (Korea) with free access to feed and water. All manipulations with animals were carried out in strict accordance with the Order of the Ministry of Health of the Russian Federation No. 199n of 1 April 2016 “Rules of Good Laboratory Practice“ and the provisions of Directive 2010/63/EU of the European Parliament and of the Council of the European Union of 22 September 2010 on the protection of animals used for scientific purposes, the requirements and recommendations of the “Guidelines for the maintenance and use of laboratory animals”.

### 5.4. Chemistry and Medicines

KI, 1% formalin solution, 0.1% histamine solution, 10% CaCl_2_ solution, adrenaline hydrochloride (AH), and the pharmaceutical preparation “Merkazolil Health”, Kharkiv, LLC “Pharmaceutical Company “Health”, Kharkiv, Ukraine. The drug diclofenac sodium at a dose of 10 mg/kg was purchased at the pharmacy. JSC, Ukraine.5.6. 

### 5.5. Experiment Design

Modeling of experimental hypothyroidism was carried out by daily oral administration of the drug “Merkazolyl Health” at a rate of 2.5 mg per 100 g of body weight for 15 days [22]. After reproducing the hypothyroidism model, all animals were divided into 3 groups (10 rats each): 

Group 1—control, received water. 

Group 2—experimental group with experimental hypothyroidism without treatment.

Group 3—correction of hypothyroidism was carried out by oral administration of an extract from the root of *R. semenovii* at a rate of 1.0 mg per 100 g of body weight. 

Group 4—hypothyroidism correction was performed with root extract of *R. semenovii* with the addition of potassium iodide at a rate of 1 μg per 100 g of a body weight. The animals received substances for correction for 28 days. All animals were on a general vivarium diet. Correction (treatment) of hypothyroidism with an extract from the root of *R. semenovii* was carried out both independently, at a rate of 1.0 mg per 100 g of a body weight and in combination with potassium iodide administered at a dose of 1 μg per 100 g of body weight for 28 days.

After the completion of the experiment, animals were sacrificed and blood was taken for hematological and biochemical analyses, which included the functional assessment of the liver, kidneys, pancreas in terms of protein, carbohydrate, lipid, and pigment types of metabolism, the presence of intoxication, and the autopsy of laboratory animals. We assessed the presence of macromorphological changes in the structure of the heart, according to check, liver, heart, and pancreas. The organs were weighed. The mass coefficients of the organs were calculated. After that, the material was fixed and placed in 10% solution of formaldehyde. To confirm hypothyroidism, serum levels of free TSH, free T4, free T3, and TG were examined using commercial kits on the automatic immunochemiluminescent analyzer “Immulite 2000XPi” (Siemens, Munich, Germany). All animal examinations were carried out in compliance with ethical principles, as well as the rules set forth in the guidelines “Rules for conducting preclinical studies, medical and biological experiments and clinical trials in the Republic of Kazakhstan” (dated 25 July 2007, No. 442).

### 5.6. Conducting Biochemical Types of Research 

After the completion of the experiment, all animals’ peripheral blood was taken for hematological and immunochemical studies into vacutainers containing the anticoagulant K3EDTA and coagulation activator with a separating gel. Hematological studies were carried out on automatic hematology analyzer Sysmex XS 550-i (Kobe, Japan). Serum TSH, free T4, free T3, and TG levels to confirm hypothyroid status on the automatic immunochemiluminescent analyzer “Immulite 2000XPi” Siemens (Munich, Germany). The blood was centrifuged for 20 min at 1000 rpm to produce plasma. The main biochemical parameters were studied: total protein, g/L, albumin g/L, urea, mmol/L, creatinine, μmol/L, uric acid, μmol/L, alkaline phosphatase, mmol/L, alanine aminotransferase, μkat/L, aspartate aminotransferase, glucose, mmol/L, cholesterol, HDL, LDL, mmol/L, and triglycerides, mmol/L. The results of the studies were recorded on the automatic biochemical analyzer BioChem-200.

### 5.7. Histological Examinations 

The thyroid gland was recorded in 10% neutral formalin, followed by standard treatment at the histological complex “Microm” and pouring into paraffin blocks. Sections with thickness of 3–4 μm were stained with hematoxylin and eosin with additional color according to the PAS (periodic acid Schiff reaction) method—hematoxylin—orange G. The degree of damage was assessed according to the following criteria: the presence of degenerative proliferative changes, the degree of fullness, and the presence of inflammatory cell infiltration.

### 5.8. Statistical Data Processing

Data processing was carried out using the program “Statistica 8.0”, making average the main indicators of systolic blood pressure. As a deviation from the average value, an average statistical error was used. The Student’s t-criterion was taken as a criterion of reliability. 

To analyze the different values between samples, the Student’s t-criterion at *p* < 0.05 was used (Statistica 12, StatSoft Inc., Tulsa, AK, USA). Processing of data obtained on the fluorimeter and plotting were performed using the capabilities of MS Excel. Atypical values based on the data were excluded from the data T-criteria. The standard error of the sample average was calculated. The plus/minus signs in the tables show the standard average error. The graphs show the average values with standard error bars. The signs * and ** indicate the validity of results at significance levels of 0.05 and 0.01, respectively (unless otherwise indicated). While determining the reliability of the difference between the indicators of compared groups, the t-confidence criterion was calculated, the value of *p* was obtained from the Student’s table of values, and the changes were considered reliable at *p* ≤ 0.05. All data were calculated in the MS Office Excel 2010 software package. Statistical data processing was also used in Statistica 10.0 with the t-criterion to assess the reliability of differences. The data are presented in the following format: average ± standard mean error (SE).

## Figures and Tables

**Table 1:** Indicators of body weight and mass coefficients of rat organs against the background of hypothyroidism after correction with extract of *R. semenovii* alone and in combination with potassium iodide.

Animal Group	Total Body Weight, g	Heart, g	Kidneys, g	Liver, g	Thyroid Gland (Together with the Trachea), g	Spleen g	Stomach, g
Control	238.5 ± 8 2	3.5 ± 0.4	2.3 ± 0.2	9.3 ± 0.6	1.1 ± 0. 01	4.6 ± 0.4	39.1 ± 3.5
Hypothy-roidism	351.4 ± 9.7 *	3.7 ± 0.6	2.3 ± 0.2	18.2 ± 0.4 *	1.4 ± 0.02 *	4.8 ± 0.4	42.7 ± 3.5
Hypothy-roidism + ERS	277.5 ± 6.7 **	3.6 ± 0.2	2.3 ± 0.2	15.5 ± 0.5 **	1.2 ± 0.01	4.6 ± 0.4	41.0 ± 2.1
Hypothy-roidism + ERS + KI	282.5 ± 18.3 ***	3.6 ± 0.6	2.2 ± 0.2	16.6 ± 0.4 ***	1.3 ± 0.02	4.8 ± 0.3	41.3 ± 2.2

Note: *—statistically significant changes in the group with hypothyroidism in relation to the control group, at *p* ≤ 0.001; **—statistically significant changes in the group with hypothyroidism + ERS in relation to the group with hypothyroidism, at *p* ≤ 0.001; ***—statistically significant changes in the group with hypothyroidism + ERS + KI in relation to the group with hypothyroidism, at *p* ≤ 0.001.

**Table 2:** Lipid metabolism indicators on hypothyroidism models after correction with pure *R. semenovii* extract and the one in combination with potassium iodide.

Group of Animals	Triglycerides, mmol/L	Total Cholesterol, mmol/L	HDL Cholesterol, mmol/L	LDL Cholesterol, mmol/L	AS
Control	0.8 ± 0.02	1.6 ± 0.03	0.9 ± 0.01	1.0 ± 0.02	0.7 ± 0.02
Hypothyroidism	0.8 ± 0.02	3.7 ± 0.9 *	1.0 ± 0.02	1.1 ± 0.03	0.8 ± 0.02
Hypothyroidism + ERS	0.7 ± 0.02	1.4 ± 0.03 **	0.9 ± 0.01	0.7 ± 0.01	0.4 ± 0.01
Hypothyroidism+ ERS + KI	0.7 ± 0.02	1.3 ± 0.03 ***	0.9 ± 0.01	0.7 ± 0.01	0.4 ± 0.01

Note: *—statistically significant changes in the group with experimental hypothyroidism in relation to the control group, at *p* ≤ 0.001; **—statistically significant changes in the group with correction of *R. semenovii* extract in relation to the group with experimental hypothyroidism, at *p* ≤ 0.001; ***—statistically significant changes in the group with correction with *R. semenovii* extract in combination with potassium iodide in relation to the group with experimental hypothyroidism, at *p* ≤ 0.001.

**Table 3:** Indicators of protein and carbohydrate metabolism on hypothyroidism models after correction with *R. semenovii* extract, alone and in combination with potassium iodide.

Group of Animals	Total Protein, g/L	Albumin g/L	Urea, mmol/L	Uric Acid, mmol/L	Creatinine, µmol/L	Glucose, mmol/L
Control	66.0 ± 2.4	29.8 ± 2.2	3.9 ± 0.3	340.8 ± 36.9	54.0 ± 3.7	6.7 ± 0.4
Hypothyroidism	69.3 ± 2.5	28.6 ± 2.1	4.3 ± 0.3	381.7 ± 41.3	58.6 ± 4.3	8.7 ± 0.5 *
Hypothyroidism + ERS	65.1 ± 2.3	29.8 ± 2.2	3.9 ± 0.3	229.0 ± 4.8 **	51.5 ± 3,8	7.4 ± 0.4
Hypothyroidism + ERS+ KI	63.2 ± 2.3	28.9 ± 2.1	3.8 ± 0.3	222.2 ± 4.0 ***	48.9 ± 3.6	6.6 ± 0.4 ***

Note: *—statistically significant changes in the group with experimental hypothyroidism in relation to the control group, at *p* ≤ 0.001; **—statistically significant changes in the group with correction of *R. semenovii* extract in relation to the group with experimental hypothyroidism, at *p ≤* 0.001; ***—statistically significant changes in the group with correction with *extract R.semenovii* in combination with potassium iodide in relation to the group with experimental hypothyroidism, at *p ≤* 0.001.

## Data Availability

Not applicable.

## Supplementary Materials

The following supporting information can be downloaded at: https://www.mdpi.com/article/10.3390/molecules27217487/s1. Figure S1. Chromatogram of tyrosol calibration solution-2 (220 nm); Figure S2. Determination of salidroside content during the flowering in the root of the plants of *R. semenovii.*
