# Supplementary material for: Antihypothyroid Effect of Salidroside
Source: Molecules. 2022 Nov 2;27(21):7487. doi: 10.3390/molecules27217487 (PMC9657580; doi:10.3390/molecules27217487)
Supplement: Supplementary file 1 [file molecules-27-07487-s001.zip › molecules-1993171-supplementary.pdf]

Supplementary Materials

Antihypothyroid Effect of Salidroside

Nazym K. Korbozova, Nataliya O. Kudrina, Nataliya A. Zhukova, Alexander E. Grazhdannikov, Irina V. Blavachinskaya, Gulnaz A. Seitimova, Timur E. Kulmanov, Tatyana G. Tolstikova * and Nina V. Terletskaya *

*Correspondence: tg_tolstikova@mail.ru (T.G.T.); teni02@mail.ru (N.V.T.); Tel.: +7-(913)-7280399 (T.G.T.); Tel.: +7-(777)-2993335 (N.T.V.)

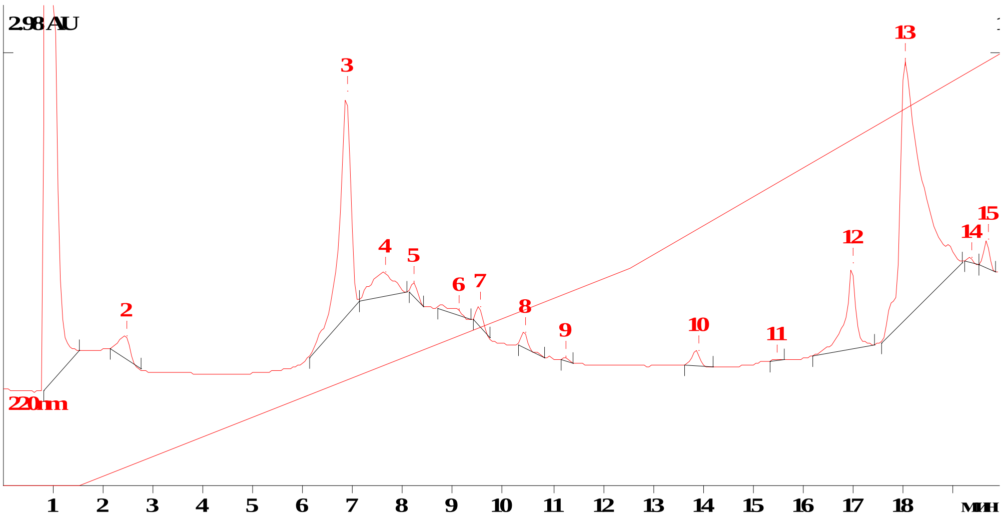

Report date: 06.12.2021 17:10:49
SAMPLE: Tyrosol 2; 3.3mg/20ml MeOH; 20mkl
Volume: 20.0 µl
COLUMN: ProntoSil-120-5-C18 AQ
Size: 2.0 x 75 mm
Part.size: 5.0 µm

Comments Tyrosol 2; in MeOH; MeOH-H3PO4 1:9-11:9-1:0 300:2500:4000
Flow: 200.00 mkl/min
Temperature: 35.0°C
Pressure: 5.0 MPa

№	Retention, mkl	Height, AU	Area, AU*mkl	Area, %	
1	180.24	18.30	586.421	63.16	
2	492.53	0.16	8.668	0.93	
3	1375.70	1.55	80.025	8.62	
4	1524.65	0.17	17.219	1.85	
5	1645.42	0.12	3.189	0.34	
6	1818.39	0.04	3.341	0.36	
7	1908.40	0.16	4.843	0.52	Tyrosol
8	2085.03	0.11	3.453	0.37	
9	2248.24	0.03	0.663	0.07	
10	2772.97	0.10	3.575	0.39	
11	3087.52	0.01	0.362	0.04	
12	3395.23	0.59	28.326	3.05	
13	3606.74	1.79	182.070	19.61	
14	3867.17	0.05	1.332	0.14	
15	3932.19	0.20	5.016	0.54	
	3981.63	23.37	928.504	100.00	

Figure S1. Chromatogram of tyrosol calibration solution-2 (220nm)

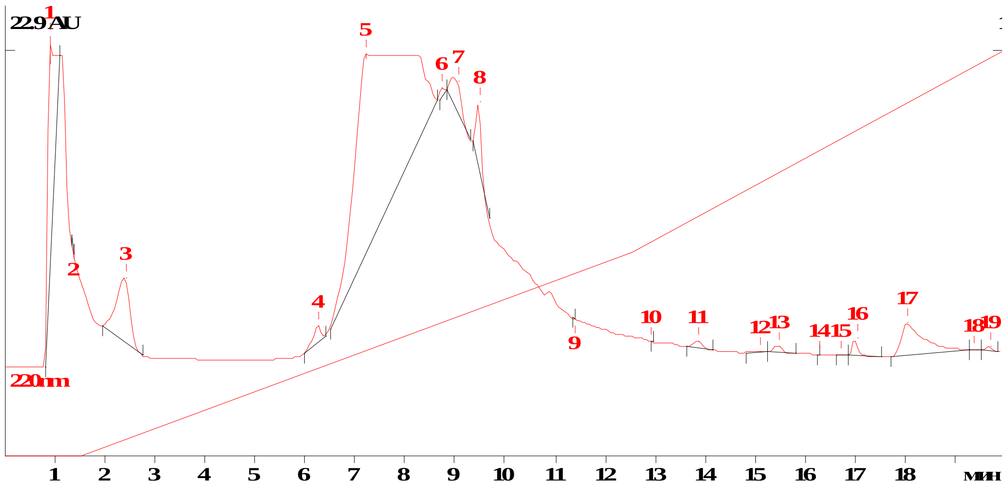

Report date: 06.12.2021 17:09:06
SAMPLE: Extr 4(upar); 169.8mg/3ml MeOH-H_2O(2:1); 20mkl
Volume: 20.0 µl
COLUMN: ProntoSil-120-5-C18 AQ
Size: 2.0 x 75 mm
Part.size: 5.0 µm
Comments Extr 4(upar); in MeOH-H2O; MeOH-H3PO4 1:9-11:9-1:0 300:2500:4000
Flow: 200.00 mkl/min
Temperature: 35.0°C
Pressure: 5.0 MPa

№	Retention, mkl	Height, AU	Area, AU*mkl	Area, %	
1	179.39	13.04	340.195	9.88	
2	270.65	-0.06	-0.329	-0.01	
3	479.76	3.59	232.402	6.75	
4	1250.88	0.86	27.442	0.80	
5	1445.62	11.01	2381.572	69.20	
6	1743.63	0.43	6.033	0.18	
7	1807.77	1.76	85.933	2.50	Tyrosol
8	1896.01	4.02	109.105	3.17	
9	2274.53	0.00	-0.012	0.00	
10	2581.62	0.00	-0.016	0.00	
11	2769.09	0.37	14.597	0.42	
12	3011.84	0.03	1.311	0.04	
13	3091.84	0.40	16.502	0.48	
14	3247.84	0.00	-0.006	0.00	
15	3335.29	0.01	0.292	0.01	
16	3396.28	1.01	25.233	0.73	
17	3604.27	1.85	192.118	5.58	
18	3869.58	0.05	1.013	0.03	
19	3931.22	0.28	7.663	0.22	
	3981.73	38.77	3441.775	100.00	

Dry residue content in extract 4: (100* 0.4367)/ 42.8450 = 1.02 %
Tyrosol content in dry part of extract 4: (100* 27.442* 3.3/20)/(80.025* 169.8/3) = 0.0999% (calibr. sol. 2 – 220nm)
Salidroside content in the dry part of extract 4:0.217%

Figure S2. Determination of salidroside content during the flowering in the root of the plants of *R. semenovii*
